# Chemical analysis, repellent, larvicidal, and oviposition deterrent activities of plant essential oils against *Aedes aegypti*, *Anopheles gambiae*, and *Culex quinquefasciatus*

**DOI:** 10.3389/finsc.2025.1582669

**Published:** 2025-05-15

**Authors:** Muhammad Ghazanfar Abbas, Muhammad Binyameen, Muhammad Azeem, Shahid Majeed, Zahid Mehmood Sarwar, Abdul Nazir, Mahar Muhammad Imran Sharif, Amna Parveen, Raimondas Mozūratis

**Affiliations:** ^1^ Laboratory of Insect Chemical Ecology, Department of Entomology, Faculty of Agricultural Sciences & Technology, Bahauddin Zakariya University, Multan, Pakistan; ^2^ Department of Chemistry, COMSATS University Islamabad, Abbottabad, Pakistan; ^3^ Department of Entomology, University of Agriculture, Faisalabad, Pakistan; ^4^ Department of Environmental Sciences, COMSATS University Islamabad, Abbottabad, Pakistan; ^5^ Laboratory of Chemical and Behavioural Ecology, Institute of Ecology, State Scientific Research Institute Nature Research Centre, Vilnius, Lithuania; ^6^ Department of Zoology, Stockholm University, Stockholm, Sweden

**Keywords:** repellence, chemical constituents, mentha spicata, control strategies, eco-friendly, gas chromatography-mass spectrometry, bioactive compounds

## Abstract

Plant-based essential oils have gained attention as a natural alternative for controlling mosquitoes due to their repellent, larvicidal and oviposition deterrent properties. We tested repellent, larvicidal, and oviposition deterrent effects of essential oils (EOs) of *Mentha* sp*icata* (L.), *Ocimum basilicum* (L.), and *Abutilon indicum* (L.) against three mosquito species (Diptera: Culicidae) including *Aedes aegypti* (L.), *Anopheles gambiae* s. l. Giles, and *Culex quinquefasciatus* Say by using contact-based technique. In screening bioassays, *M.* sp*icata I*, *M.* sp*icata II*, *O. basilicum I*, *O. basilicum II*, and *A. indicum* EOs showed higher repellency against *Cx. quinquefasciatus* as compared to *Ae. aegypti* and *An. gambiae* when tested at 33.3 μg/cm^2^. In time-span bioassays performed at 33.3 μg/cm^2^, EO of *M.* sp*icata I* exhibited 100% repellence up to 45, 30, and 75 min against *Ae. aegypti*, *An. gambiae*, and *Cx. quinquefasciatus*, respectively. Interestingly, at this tested dose, *M.* sp*icata I* and *M.* sp*icata II* showed higher repellence compared to DEET against *Ae. aegypti* and *Cx. quinquefasciatus* after 45 and 75 min, respectively. Their repellency was observed up to 150 and 210 min against *Ae. aegypti* and *Cx. quinquefasciatus*, respectively. In larvicidal bioassays, *M.* sp*icata I* EO proved more toxic against 2^nd^ instar larvae of *Ae. aegypti*, *An. gambiae*, and *Cx. quinquefasciatus* (LC_50_ = 11.0, 42.9, and 12.6 mg/L, respectively) compared to other tested EOs. In oviposition bioassays, *M.* sp*icata I* exhibited the highest activity, showing 60%, 46%, and 79% oviposition deterrence against *Ae. aegypti*, *An. gambiae*, and *Cx. quinquefasciatus*, respectively, tested at a dose of 600 µg/cm^2^. Major compounds of *M.* sp*icata I*, *M.* sp*icata II*, *O. basilicum I*, and *O. basilicum II* EOs were piperitenone oxide (38.8%), piperitone oxide (35.4%), estragole (55.3%), and linalool (43.8%), respectively. In conclusion, *M.* sp*icata* EO could be used to control mosquitoes and their bites.

## 1 Introduction

Mosquito-borne diseases are widespread in tropical and subtropical regions of the world, ranging from asymptomatic to severe, and can even be fatal (1). Chikungunya, for example, can cause chronic joint pain that can last for years. Similarly, Zika infection has been associated with neurological disorders and fatal abnormalities during pregnancy (2). Malaria can have a devastating impact on the socioeconomic development of affected communities by lowering workforce productivity and raising healthcare costs. Children are especially susceptible to these vector-borne diseases. Filariasis affects over 120 million people worldwide, causing debilitating symptoms like elephantiasis (3). Every year, over one million people worldwide die as a result of mosquito-borne diseases (4, 5). Keeping in mind the harmful effects of diseases associated with mosquitoes, there is a need to control the population of mosquitoes and use personal protection means against their biting.

Synthetic insecticides such as temephos, deltamethrin, metofluthrin, acetamiprid, and cypermethrin effectively control mosquitoes (6–8). However, using these traditional insecticides can have negative consequences, including resurgence, resistance development, residual effects on the environment, and negative impacts on non-target organisms (9). Insecticides can harm humans, causing reproductive, carcinogenic, and endocrine problems (10, 11). Besides controlling mosquitoes through insecticides, personal protection is considered a suitable approach to prevent the bite of mosquitoes. N, N-diethyl-m-toluamide (DEET) and IR3535 are synthetic repellent compounds used against blood-sucking insects and effectively deter mosquitoes. However, continuous and excessive use of these synthetic repellents could harm human health, i.e., swelling, eye irritation and rashes (12–14). The alternative approach could be a way forward to combat mosquitoes and mosquito-borne diseases.

As a result, scientists have been focused on developing plant-based pest-control solutions. The products extracted from plants have proved effective in controlling insect pests for an extended period without harming the environment (15–17). Essential oils (EOs) derived from plants have been traditionally used in controlling insect pests (18, 19). These have a considerable share of the pesticide market, accounting for around $700 million with 45,000 tons of the world’s total pesticide output (20). EOs are being considered alternatives to synthetic insecticides in controlling mosquitoes due to their selective action in controlling target pests, as well as their minimal effects on non-target organisms and high environmental degradations (21). There are a few plant-based mosquito repellents available in the market. For example, the United States Environmental Protection Agency has approved Java citronella oil as a blood-sucking insect repellent (22). The *p*-menthane-3,8-diol (PMD) is a monoterpene, another plant-based natural product used as a mosquito repellent. It is a spent product of the distillation of leaves of the Australian lemon-scented gum tree, *Corymbia citriodora* (Hook.) (Myrtales: Myrtacea), commonly known by the synonym *Eucalyptus citriodora*. The U.S. Centers for Disease Control and Prevention (CDC) endorsed two non-DEET mosquito repellents, including PMD, in April 2005 (20, 23).

In our previous study, we screened seven EOs for repellent activity against *Aedes aegypti* L. (Diptera: Culicidae) mosquitoes and revealed that the EO of *Mentha* sp*icata* L. (Lamiales: Lamiaceae) was the most efficient repellent (24). In the proposed study, two different chemotypes of *M.* sp*icata* and *Ocimum basilicum* L (Lamiales: Lamiaceae) were used to test their bioactivity against three different mosquito species. Although several studies have documented the mosquito repellent and larvicidal properties of *M.* sp*icata* and *O. basilicum*, there is a notable gap in the biological activities of their various chemotypes. While the carvone and piperitenone oxide chemotypes of M. spicata are extensively reported (25–30) the piperitone oxide chemotype of *M.* sp*icata* is seldom mentioned. Besides *M.* sp*icata* and *O. basilicum*, *Abutilon indicum* L. was also studied. To our best knowledge, only a few publications reported the larvicidal effect of EOs derived from Indian mallow *Abutilon indicum* (L.) (Malvales: Malavaceae) (31–33), while repellent and oviposition deterrent activities remain unknown. Moreover, to evaluate the potential use of a natural product, its efficiency and longevity of action are commonly compared with the most efficient positive control. Keeping in mind the importance of EOs in controlling mosquitoes, the current study was performed to comprehensively evaluate the most important bioactivities concerning mosquito control, i.e., repellent, larvicidal, and oviposition deterrent activities of selected EOs against three species of mosquitoes (Diptera: Culicidae): yellow fever mosquito, *Ae. aegypti*, *Anopheles gambiae* s. l., and southern house mosquito *Culex quinquefasciatus* Say.

## 2 Materials and methods

### 2.1 Collection and maintenance of plant material

The fresh aerial parts of selected plants were hand-picked in the flowering season. The samples of *M.* sp*icata* and *O. basilicum* were collected from different locations in the district of Bhakkar, Pakistan whereas a sample of *A. indicum* was collected from Multan, Pakistan (**Table 1**). Some plants from each sample were isolated, placed in blotting sheets, and pressed using a standard process. Each plant species specimen was mounted on a standard herbarium sheet and deposited in the herbarium of the Department of Environmental Sciences, COMSATS University Islamabad, Abbottabad Campus, Abbottabad, Pakistan for record, and a voucher number was assigned (34). The identification of the plants was authenticated by a plant taxonomist at the Department of Environmental Sciences, COMSATS University Islamabad, Abbottabad Campus, Abbottabad, Pakistan. The plant samples to be used for EO extraction were gently washed with tap water and then rinsed with distilled water. The plant material was spread on a white cotton cloth in a shady area and air dried using a ceiling fan. All plant samples were processed in the same way but in separate rooms. The shade-dried plant material was stored in airtight poly bags at room temperature for about 2 weeks until used for EOs extraction.

**Table 1:** Plant material used in the study and percentage yield of EOs.

Plant name	Voucher No	Family	Collection coordinates	Elevation (m)	Yield (%)
*Mentha* sp*icata I*	CUHA-472-1	Lamiaceae	31°39’17.9”N 71°11’58.3”E	172	0.44
*Mentha* sp*icata II*	CUHA-472-2		31°57’31.6”N 71°20’29.9”E	185	0.84
*Ocimum basilicum I*	CUHA-470-1		32°05’50.9”N 71°26’22.0”E	190	1.1
*Ocimum basilicum II*	CUHA-470-2		31°57’31.6”N 71°20’29.9”E	185	1.01
*Abutilon indicum*	CUHA-471	Malavaceae	30°16’44.8”N 71°31’12.3”E	124	0.02

### 2.2 Extraction of EOs

The steam distillation method was used to extract EOs from the collected plant material using a Clevenger-type apparatus, as described in the previous study (24). A stainless-steel vessel was loaded with plant material (300g), and two litres of distilled water were added to the bottom of the vessel. Water had no direct contact with the plant material. The distillation vessel was heated by using an electric hotplate. Volatile compounds released from the plant material and steam were cooled using a condenser fitted on the head of the vessel, and the distillate was collected in a separating funnel for three hours. The EO layer formed above the water layer was decanted and dried over anhydrous MgSO_4_. The extracted EO was weighed, and the percentage yield was calculated using the dry mass of the plant. The samples of EOs were stored at -20 °C until used for bioassays and chemical analysis.

### 2.3 Rearing of mosquitoes

*Ae. aegypti*, *An. gambiae* s. l., and *Cx. quinquefasciatus* mosquitoes were reared in the laboratory using methods described in previous studies (24, 35–38). The mosquitoes at the larval stage were obtained from the Punjab Health Department, Multan, Pakistan. Larvae were placed in a plastic container (20 × 16 × 4 cm) filled with 1 L water and fed with fish food (Osaka green fish food, India) containing 3% crude fat, 4% crude fibre, and 28% crude protein. Pupae were collected daily from the larval container and transferred to plastic cups containing 200 mL of tap water. The plastic cups were placed in Plexi-glass cages (30 × 30 × 30 cm) for the emergence of adults. Cotton soaked with 10% sucrose solution was placed in cages as an adult diet. After 4–5 days, females (*Ae. aegypti* and *Cx. quinquefasciatus*) were fed blood from a constrained pigeon placed in the adult cage while *An. gambiae* were fed on human arm blood. Wax paper was wrapped on the inner walls of the plastic jar, filled with water and placed in the adult cage for oviposition. After oviposition, the wax paper with eggs was transferred to the larval container with 1000 mL of tap water for hatching. The rearing process continued until enough adults and larvae were obtained for the repellence, oviposition and larvicidal bioassays, respectively. The rearing of three mosquito species was carried out in separate rooms. All rearing was carried out in a controlled room maintained at 25 ± 2°C for *Ae. aegypti* and *Cx. quinquefasciatus* while for *An. gambiae* room was maintained at 30 ± 2°C. Relative humidity was maintained at 80 ± 10% with a photoperiod of 12h:12h light:dark.

### 2.4 Mosquito repellency bioassay

A human bait technique was used to test the repellence potential of EOs against *Ae. aegypti*, *An. gambiae* s. l., and *Cx. quinquefasciatus* females (24, 35, 39). The positive control DEET (99% purity, Sigma-Aldrich, St. Louis, MO, USA) and EOs solutions, 1% (10 mg/mL) and 10% (100 mg/mL), were prepared by dissolving the respective substances in absolute ethanol (Daejung, Korea). Ethanol was used as a negative control in repellency bioassays. Twenty mated and blood-starved 4–5 days old female *Ae. aegypti* (strains 10) were released from the laboratory-reared colony in the experimental cage (30 × 30 × 30 cm) about 12 h before the start of the repellency bioassay. The hands of the subjects (2 volunteers) were washed with scent-free liquid soap and allowed to dry for about 10 min before starting the bioassay. Plastic gloves were used to cover the subject’s hand except for the 30 cm^2^ circular area on the dorsal side of the hand. A 100 μL aliquot solution of the test substance (1% or 10% equivalent to 33.3 µg/cm^2^ and 333 µg/cm^2^, respectively) or pure solvent as a negative control was evenly applied on the exposed area of the hand and dried in air for three min before exposing the hand to *Ae. aegypti* females. The subject’s hand was exposed to the females in the experimental cage, and mosquito landings were counted for 5 min. The experiment was repeated randomly five times for both the test samples and the negative control. The same procedure was followed to evaluate the repellency of EOs against *An. gambiae* and *Cx. quinquefasciatus*. The human volunteers were informed about the test procedure, and consent was obtained before conducting repellence bioassays. The repellency percentage was calculated using the formula: percentage repellency = [(Mc – Mt)/Mc] ×100, where Mc is the number of mosquito landings on the negative control (solvent) treated hand and Mt is the number of mosquito landings on the test substance treated hand. All volunteers followed standardised procedures to minimise the variability.

### 2.5 Time span bioassays

Plant EOs that showed at least 50% repellence were further investigated to determine their repellent longevity. Time-span repellent bioassays were performed by following the same protocol as mentioned above in the repellency bioassay, except for the exposure of the same treated hand to the females of *Ae. aegypti*, *An. gambiae* s. l., and *Cx. quinquefasciatus* for 5 min after each 15 min interval until the number of landings on control and treatment didn’t differ significantly. Time span bioassays were conducted using test samples at the dosages of 33.3 μg/cm^2^ and 333 μg/cm^2^. The experiments were repeated five times, and fresh female mosquitoes were employed for each replicate. A repellency bioassay for each mosquito species was conducted in separate climate-controlled rooms.

### 2.6 Larvicidal bioassays

Larvicidal bioassays against 2^nd^ instar larvae of *Ae. aegypti*, *An. gambiae*, and *Cx. quinquefasciatus* were conducted through a modified protocol described in previous studies (38, 40). Briefly, ice cube trays (50 mL well size) were cleaned with tap water, and each well of an ice cube tray was filled with 20 mL of water. Fifteen 2^nd^ instar larvae of *Cx. quinquefasciatus*, or *Ae. aegypti, or An. gambiae* were added through a pipet to each well of the ice cube tray. DMSO was used to dilute the tested EOs, and 50 µL of the resulting solution was added to each well, with a final concentration ranging from 6.25 mg/L to 1600 mg/L. The 2^nd^ instar larvae of tested mosquitoes were exposed to EOs or DMSO for 24 and 48 hours to assess their susceptibility. A fish diet was used to feed larvae during the exposure time. The larvae that did not show movement after the exposure period were considered dead. DMSO was used as a negative control, and its concentration in test media never exceeded 0.25%. Chlorpyriphos was used as the positive control. Each experiment was repeated at least six times to ensure the reliability and reproducibility of the results.

### 2.7 Oviposition deterrence

The oviposition deterrence bioassay was conducted by adopting a method described by previous studies (38, 41). Briefly, sixty 5–7 days old and blood-fed female mosquitoes were released in a bioassay cage. Two plastic cups filled with 100 mL of distilled water were placed diagonally in the corners of the bioassay cage. One cup was a test treatment, while the other was a control. An aliquot of 600 µL of 1% or 10% ethanolic solution of an EO (w/v) was evenly sprayed on half of the wax paper strip (10 × 20 cm), air dried for 2 min, and then wrapped along the inner walls of the water-filled plastic cup in such a way that the EO treated area (10 × 10 cm) remained above the water level. The overall concentration on the treated wax paper was 60 µg/cm^2^ for 1% and 600 µg/cm^2^ for 10% ethanolic solution of an EO. In the control cup, the solvent-treated filter paper was wrapped in the same way described for the test cup. After applying the sample or solvent, the cups were left outside the cages for 5 min so that the solvent could evaporate before the commencement of the experiment. The control and sample-treated cups were left in the adult mosquito cage for 48 hours for oviposition. Afterwards, the eggs laid in each cup were counted. The positions of the control and test cups were changed randomly to avoid the position effects on oviposition. We conducted oviposition tests in each experiment, five times using a fresh mosquito population.

### 2.8 Chemical analysis of EOs

EOs that showed about 50% or higher repellence against all tested species of mosquitoes were analysed using a Hewlett Packard gas chromatograph connected to a mass spectrometer (GC–MS) by adopting the method described in earlier studies (24, 38). The GC had a 30 m capillary column (DB-5, Agilent Technologies Inc., Santa Clara, CA, USA) with a 0.25 mm internal diameter and a stationary phase film thickness of 0.25 µm. The GC injector temperature was maintained isothermally at 235°C throughout the sample analysis. The GC oven temperature was programmed as follows: initial temperature of 40°C for 2 min, then increased to 240°C at a rate of 4°C per min, and finally maintained at 240°C for 8 min. Helium was used as the mobile phase at a constant flow rate of 1 mL/min through the column. An aliquot of 1 µL of dilute EO solution was injected into the GC, and the injector was operated in a splitless mode for 30 sec. The MS was operated using the following parameters: an electron energy of 70 eV for ionisation, an ion source temperature of 180°C, and a mass spectrum scan range of 30–400 m/z. The total ion chromatogram was used to calculate the percent composition of compounds in EOs. A solution of a series of straight-chain alkanes (C_9_-C_24_) was injected into the GC-MS using the same parameters as the EOs analyses. The retention times of unknown compounds and alkanes were used to calculate the retention indices of separated compounds. Mass spectra and retention indices of separated compounds were initially compared to those available in the NIST-2008 MS library and webbook NIST online library to identify the separated compounds. Finally, identifications were verified by injecting available pure standard compounds purchased from Sigma–Aldrich (St. Louis, MO, USA) and Alfa Aesar (Haverhill, MA, USA).

### 2.9 Statistical analysis

To evaluate the statistical difference between the repellence and oviposition deterrent activity data of different EOs, one-way ANOVA was used, followed by the Tukey test at a significant threshold of alpha = 0.05 for pairwise comparisons of group means. The statistical analysis was performed on the Statistica 8.1 software version 14.0.1.25 (TIBCO Software Inc, Palo Alto, CA, USA). The observed larvicidal activities (LC_50_) were determined using the computer software SPSS 20 (IBM, USA). The lethal concentration estimates for tested essential oils were considered significantly different (*p* < 0.05) from the baseline essential oil if confidence limits for relative median potency ratios did not overlap with the value 1 (35, 38).

## 3 Results

### 3.1 Yield (%) of EOs

The aerial parts of *O. basilicum* I contained the highest amount of EO, yielding 1.10%, while the leaves of *A. indicum* contained the lowest amount of EO, i.e. 0.02% (**Table 1**).

### 3.2 Screening repellent bioassays

**Figure 1:**
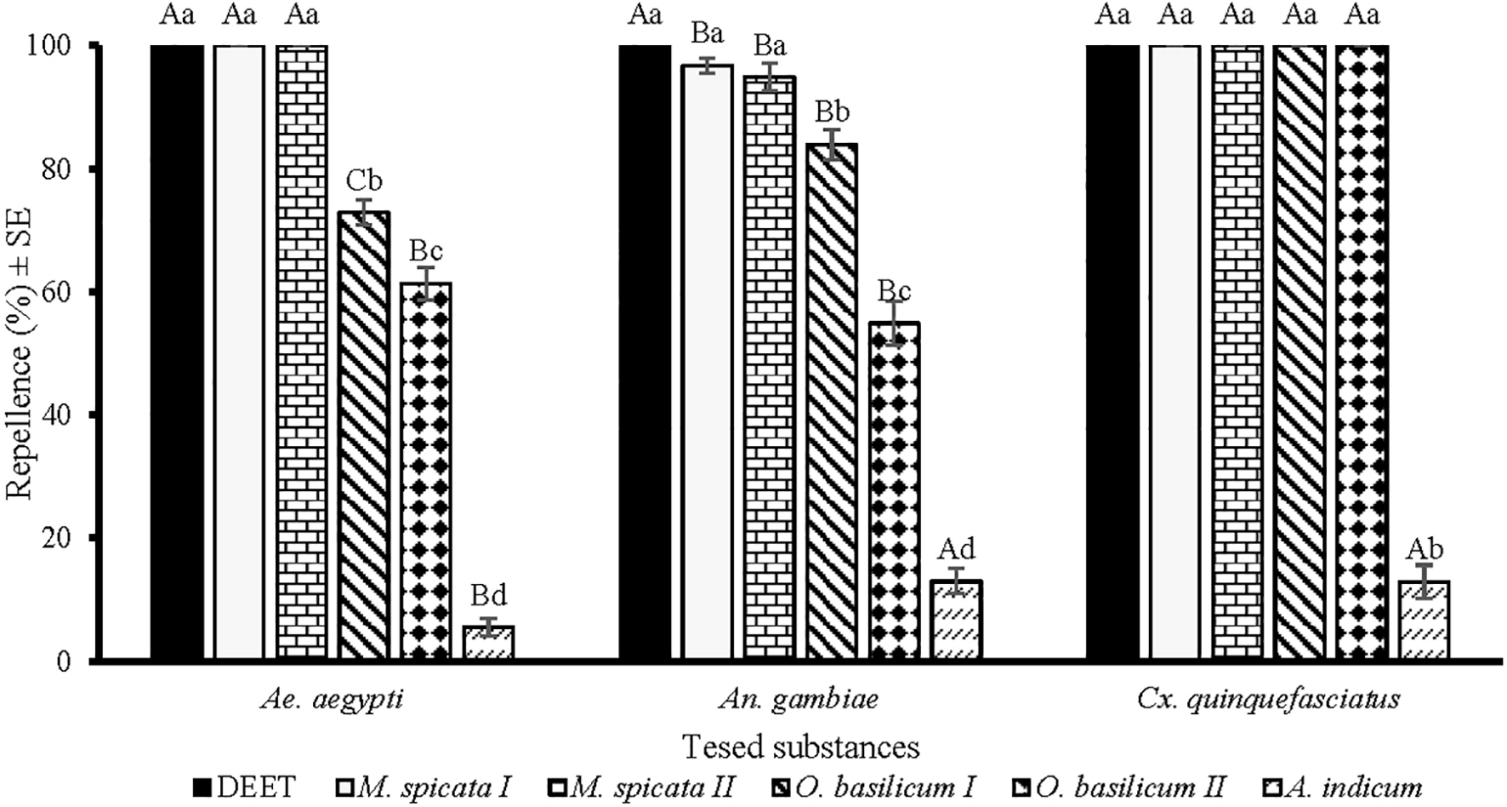
Repellency of positive control DEET and EOs at 33.3 µg/cm^2^ against *Aedes aegypti*, *Anopheles gambiae* s. l., and *Culex quinquefasciatus* mosquito females. Lower case letters above the columns represent significant differences (ANOVA *post-hoc* Tukey, *p* < 0.05) among tested substances against each mosquito species, while upper case letters represent the significant difference (ANOVA *post-hoc* Tukey, *p* < 0.05) among different species of mosquitoes towards a particular test substance. Error bars represent the standard error (n =5).

There were significant differences in the repellency of *M.* sp*icata I*, *M.* sp*icata II*, *O. basilicum I*, *O. basilicum II*, and *A. indicum* EOs against females of *Ae. aegypti* (*df* = 5, *F* = 1048, *p* < 0.001), *An. gambiae* (*df* = 5, *F* = 399, *p* < 0.001), and *Cx. quinquefasciatus* (*df* = 5, *F* = 1741, *p* < 0.001) at a tested dose of 33.3 µg/cm^2^ (**Figure 1**). DEET showed a similar repellency (100%) against *Ae. aegypti*, *An. gambiae*, and *Cx. quinquefasciatus*. Importantly, *M.* sp*icata I* and *M.* sp*icata II* EOs proved most effective and showed similar patterns of repellence against all tested mosquito species (**Figure 1**). *M.* sp*icata I* and *M.* sp*icata II* EOs displayed 100% repellency against *Ae. aegypti* and *Cx. quinquefasciatus* while against *An. gambiae* displayed 96% and 94% repellence, respectively. *O. basilicum I* and *O. basilicum II* EOs provided complete protection (100%) against *Cx. quinquefasciatus*. EO distilled from *A. indicum* revealed the least repellence against tested species of mosquitoes as compared to other tested EOs. Comparatively, more repellence of tested EOs was observed against *Cx. quinquefasciatus* compared to *Ae. aegypti* and *An. gambiae* (**Figure 1**).

In screening bioassays, *M.* sp*icata I*, *M.* sp*icata II*, *O. basilicum I*, and *O. basilicum II* EOs showed more than 50% repellence against all tested species of mosquitoes and were further tested to evaluate the maximum period of repellency.

### 3.3 Time-span repellency of tested substances against Ae. aegypti

**Figure 2:**
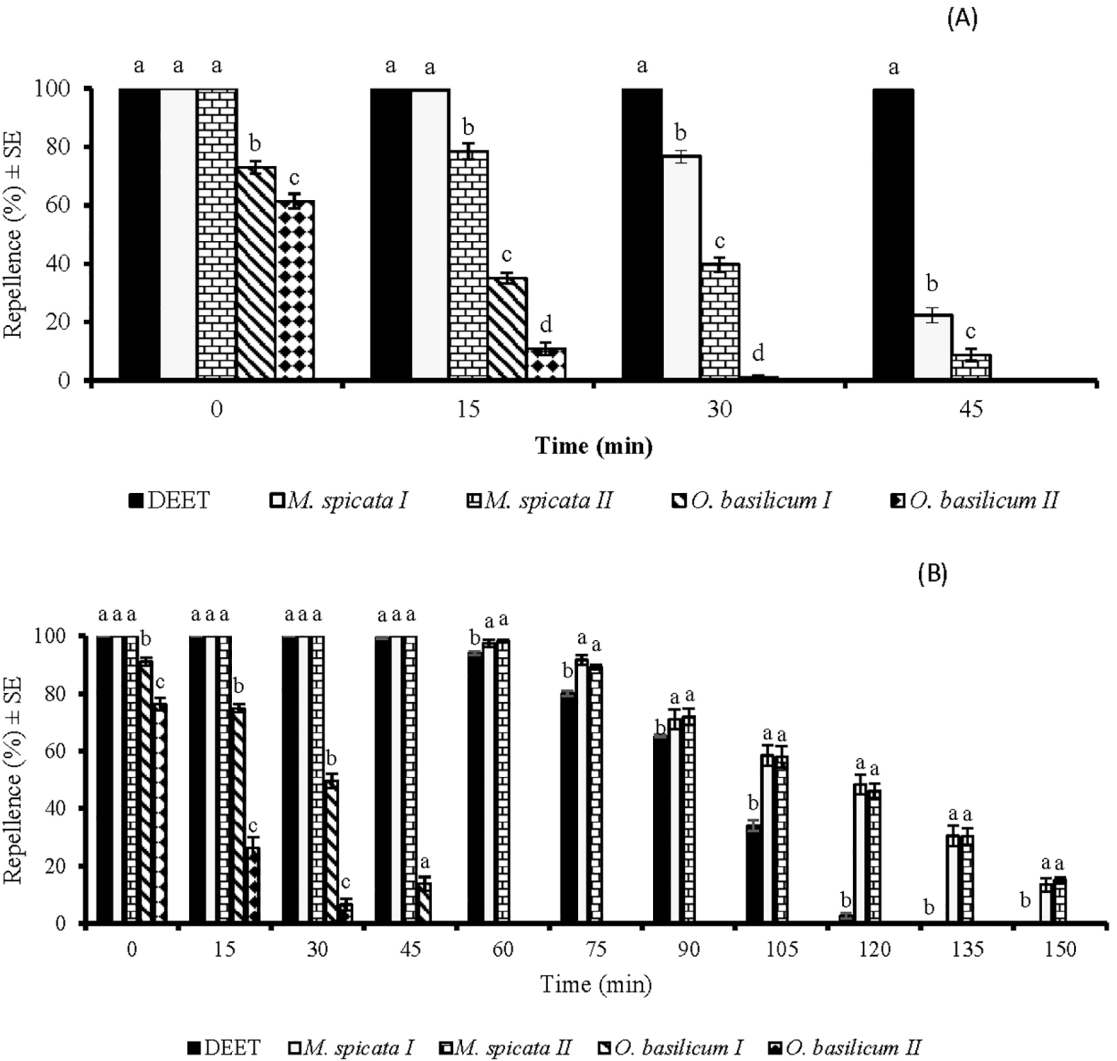
Time span repellent effect of DEET and four EOs against *Aedes aegypti* females at doses of 33.3 µg/cm^2^ **(A)** and 333 µ g/cm^2^ **(B)**. Different letters indicate significant differences (*p* < 0.05) in repellency between different tested samples within each time interval, according to the ANOVA *post-hoc* Tukey test. “SE” stands for standard error (n = 5).

In time span bioassays, statistical data analysis revealed a significant impact of EOs repellency against *Ae. aegypti* at 33.3 µg/cm^2^ (*df* = 4, *F* = 254, *p* < 0.001). *M.* sp*icata I* and *M.* sp*icata II* EOs displayed 100% repellency against *Ae. aegypti* when tested immediately after application at a dose of 33.3 µg/cm^2^ (**Figure 2A**). However, their repellent potential decreased over time and reached 22% and 8%, respectively, at 45 min post-treatment. EOs of *O. basilicum I* and *O. basilicum II* showed 71% and 61% repellency, respectively, at 33.3 µg/cm^2^, and their repellency was observed only for up to 15 min (**Figure 2A**).

There was a significant difference (*df* = 4, *F* = 131, *p* < 0.001) in the repellent activities of EOs against *Ae. aegypti* tested at a dose of 333 µg/cm^2^. *M.* sp*icata I* and *M.* sp*icata II* EOs displayed complete protection against *Ae. aegypti* for up to 45 min (**Figure 2B**). Interestingly, these EOs exhibited higher repellency (*p* < 0.05) against *Ae. aegypti* at this tested dose compared to DEET after 45 min post-treatment, and their repellency was observed for up to 150 min post-treatment (**Figure 2B**). EOs of *O. basilicum I* and *O. basilicum II* showed 91% and 76% repellency, respectively, against *Ae. aegypti* at 333 µg/cm^2^, when applied immediately after application. However, both EOs showed repellence only for 30 and 45 min, respectively and later, no repellency was observed against *Ae. aegypti* (**Figure 2B**).

### 3.4 Time-span repellency of tested substances against An. gambiae

**Figure 3:**
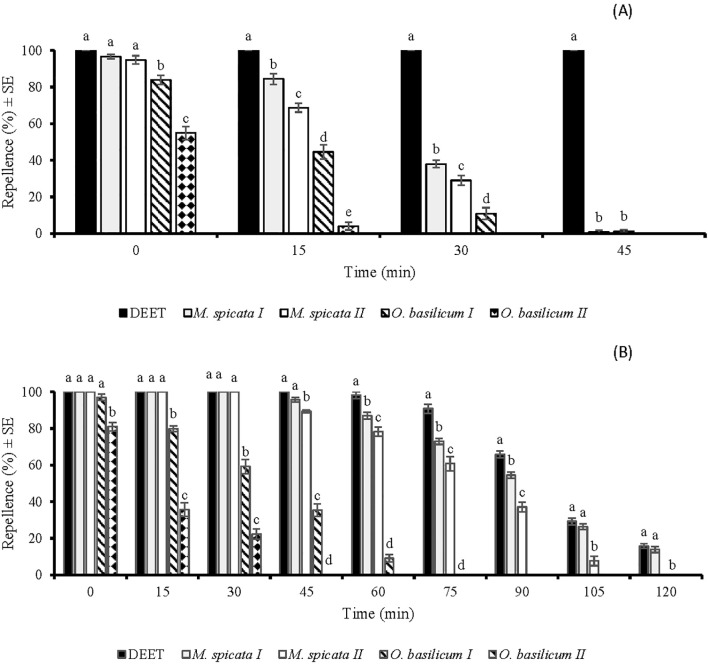
Time spans repellent effect of DEET and four EOs against *Anopheles gambiae* s. l. females at doses of 33.3 µg/cm^2^ **(A)** and 333 µg/cm^2^ **(B)**. Different letters indicate significant differences (*p* < 0.05) in repellency between samples within each time interval according to the ANOVA *post-hoc* Tukey test. “SE” stands for standard error (n =5).

There was a significant difference in the repellency of tested EOs against *An. gambiae* at 33.3 µg/cm^2^ (*df* = 4, *F* = 113, *p* < 0.001) (**Figure 3A**). *M.* sp*icata I*, *M.* sp*icata II* EOs, and DEET showed 100% repellence against *An. gambiae*, when tested immediately after application of 33.3 µg/cm^2^ (**Figure 3A**). However, later repellency of *M.* sp*icata I* and *M.* sp*icata II* EOs decreased to 37% and 34%, respectively, at 30 min post-treatment. Comparatively, *O. basilicum II* EO showed the least repellence against *An. gambiae* at 33.3 µg/cm^2^ (**Figure 3A**).

At a higher tested dose (333 µg/cm^2^), a significant difference (*df* = 4, *F* = 69, *p* < 0.001) was observed in the repellency of tested EOs against *An. gambiae* (**Figure 3B**). *M.* sp*icata I* and *M.* sp*icata II* showed complete protection similar to DEET against *An. gambiae* for up to 45 and 30 min, respectively. *M.* sp*icata I* EO and positive control showed similar repellence against *An. gambiae* at 0, 15, 30, 45, 105, and 120 min post-treatment. *O. basilicum I* and *O. basilicum II* EOs exhibited an active time-span repellence against *An. gambiae* for up to 60 and 30 min, respectively (**Figure 3B**).

### 3.5 Time-span repellency of tested substances against *Cx. quinquefasciatus*

**Figure 4:**
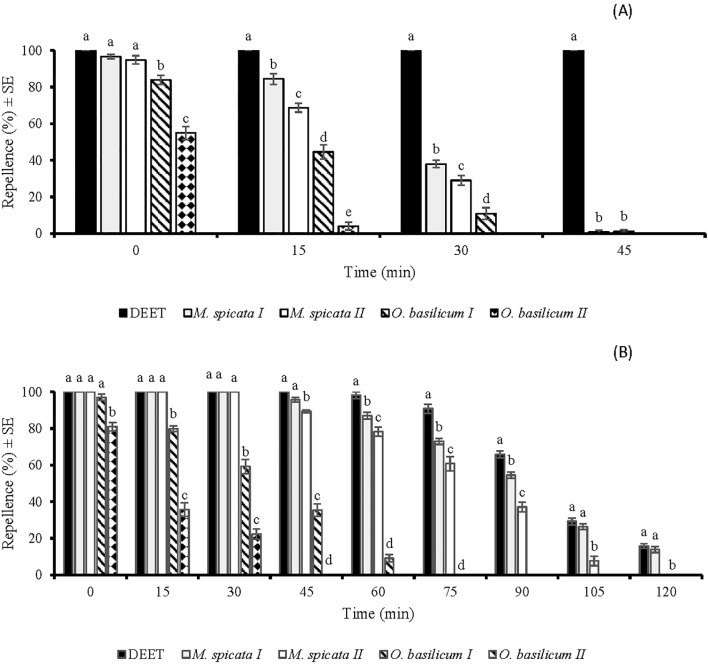
Time spans repellent effect of DEET and four EOs against *Culex quinquefasciatus* females at doses of 33.3 µg/cm2 **(A)** and 333 µg/cm2 **(B)**. Different letters indicate significant differences (*p* < 0.05) in repellency between different substances within each time interval according to the ANOVA *post-hoc* Tukey test. “SE” stands for standard error (n =5).

The repellency of all tested substances (EOs and DEET) was a significant difference (*p* < 0.05) at each tested time span except for the immediate post-treatment application of (*p* > 0.05). All the tested EOs showed 100% repellence against *Cx. quinquefasciatus* when tested immediately after application at a tested dose of 33.3 µg/cm^2^ (**Figure 4A**). The EOs of *M.* sp*icata I* and *M.* sp*icata II* exhibited complete protection (100%) against *Cx. quinquefasciatus* up to 60 and 35 min, respectively. Both these EOs showed repellence against *Cx. quinquefasciatus* up to 150 min and 135 min, respectively. *O. basilicum I* and *O. basilicum II* EOs showed 100% repellence against *Cx. quinquefasciatus* when observed immediately after application. However, at 30 min post-treatment, their repellency against *Cx. quinquefasciatus* decreased to 39% and 19%, and later, no repellency was observed (**Figure 4A**).

The application of tested EOs at higher dose (333 µg/cm^2^) extended the active time span of *M.* sp*icata I*, *M.* sp*icata II*¸ *O. basilicum I*, *O. basilicum II*. DEET, *M.* sp*icata I*, and *M.* sp*icata II* EOs showed 100% repellency for 75 min, 120 min, and 105 min, respectively. Afterwards, the efficiency of these substances started to decrease at different rates (*p* < 0.05). Importantly, EOs of *M.* sp*icata I* and *M.* sp*icata II* showed more repellence against *Cx. quinquefasciatus* after 75 min post-treatment as compared to other tested substances. Repellent effects of *M.* sp*icata I* and *M.* sp*icata II* against *Cx. quinquefasciatus* were observed up to 210 min, while repellent effects of *O. basilicum I* and *O. basilicum II* EOs were observed up to 120 and 105 min respectively (**Figure 4B**).

### 3.6 Larvicidal effects of EOs

All the tested EOs showed larvicidal effects against 2^nd^ instar larvae of *Ae. aegypti*, *An. gambiae*, and *Cx. quinquefasciatus* (**Tables 2**, **3**). Positive control (chlorpyriphos) exhibited higher larvicidal effects (p < 0.05) against all tested species of mosquitoes as compared to all tested EOs. Larvae of *Ae. aegypti* proved significantly more susceptible to *M.* sp*icata I* and *O. basilicum I* compared to *M.* sp*icata II*, *O. basilicum II*, and *A. indicum* EOs at 24 and 48 h of exposure (**Table 2**, **3**). There was no significant difference (p > 0.05) in the larvicidal effects of *M.* sp*icata I* and *O. basilicum I* EOs against *Ae. Aegypti* thus showed LC_50_ values of 15.7 mg/L and 18.5 mg/L respectively after 24 h exposure. In the case of *An. gambiae*, *M.* sp*icata I* showed high toxic effects (LC_50_ = 52.1 mg/L (ppm) at 24 h exposure and LC_50_ = 42.9 mg/L at 48 h) against larvae of *An. gambiae* while *A. indicum* showed the least toxic effects compared to other tested EOs. In the case of *Cx. quinquefasciatus*, the LC_50_ value of *M.* sp*icata I* was 18.2 mg/L at 24 h exposure, which decreased to 12.6 mg/L after 48 h of larvae exposure. Overall, *M.* sp*icata I* showed higher while *A. indicum* showed the least larvicidal effects against *Ae. aegypti*, *An. gambiae*, and *Cx. quinquefasciatus* compared to *M.* sp*icata II*, *O. basilicum I*, and *O. basilicum II* at both tested time (**Tables 2**, **3**).

**Table 2:** Toxicity of tested substances against 2nd instar mosquito larvae after exposure of 24 h.

*Ae. aegypti*				
Tested substances	*LC_50_ (mg/L)	#lower limit	upper limit	χ^2^
*M.* sp*icata I*	15.7 b	10.6	22.8	1.9
*M.* sp*icata II*	25.1 c	13.1	44.3	0.5
*O. basilicum I*	18.5 b	12.6	26.8	0.9
*O. basilicum II*	37.6 d	26.2	53.7	0.7
*A. indicum*	1241.5 e	816.9	1950.2	0.3
Chlorpyriphos	2.1 a	1.01	5.2	0.4
*An. gambiae*				
*M.* sp*icata I*	52.1 b	45.9	151.3	1.4
*M.* sp*icata II*	185.9 d	140.4	381.9	2.1
*O. basilicum I*	83.9 c	65.4	19.8	1.2
*O. basilicum II*	213.6 d	149.7	395.2	1.4
*A. indicum*	912.2 e	512.1	2299.7	0.8
Chlorpyriphos	2.0 a	0.9	6.1	0.3
*Cx. quinquefasciatus*				
*M.* sp*icata I*	18.2 b	19.4	50.7	1.5
*M.* sp*icata II*	77.2 d	46.9	148.2	2.1
*O. basilicum I*	25.3 c	19.2	49.5	1.7
*O. basilicum II*	81.4 d	56.1	162.4	2.1
*A. indicum*	1123 e	771.1	5824.1	1.9
Chlorpyriphos	2.2 a	0.7	7.5	0.4

*LC_50_ Lethal concentration to kill 50% larvae of mosquitoes. χ^2^ (Chi-square). # 95% confidence limits (lower and upper) for LC_50_. LC_50_ values with different letters indicate significant differences based on the relative median potency analysis of EOs against each larvae type independently.

**Table 3:** Toxicity of tested substances against 2nd instar mosquito larvae after exposure of 48 h.

*Ae. aegypti*				
Tested substances	*LC_50_ (mg/L)	#lower limit	upper limit	χ^2^
*M.* sp*icata I*	11.0 b	7.5	18.9	0.9
*M.* sp*icata II*	22.1 c	15.4	39.6	1.4
*O. basilicum I*	13.5 b	9.3	19.4	1.2
*O. basilicum II*	26.3 c	20.1	41.8	0.7
*A. indicum*	921.4 d	500.3	2059.8	1.2
Chlorpyriphos	1.8 a	0.78	3.78	0.2
*An. gambiae*				
*M.* sp*icata I*	42.9 b	29.5	87.4	2.4
*M.* sp*icata II*	147.4 d	94.1	261.3	2.1
*O. basilicum I*	65.7 c	34.9	115.3	1.5
*O. basilicum II*	178.3 e	112.4	319.3	1.4
*A. indicum*	740.9 f	492.7	1422.6	0.6
Chlorpyriphos	1.7 a	0.5	4.8	0.2
*Cx. quinquefasciatus*				
*M.* sp*icata I*	12.6 b	7.2	26.3	1.8
*M.* sp*icata II*	53.9 d	33.4	131.5	1.7
*O. basilicum I*	20.9 c	14.6	47.9	1.6
*O. basilicum II*	55.8 d	39.6	121.3	2.3
*A. indicum*	821.7 e	499.3	2071.8	1.2
Chlorpyriphos	1.4 a	0.3	7.7	0.5

*LC_50_ Lethal concentration to kill 50% larvae of mosquitoes. χ^2^ (Chi-square). # 95% confidence limits (lower and upper) for LC_50_. LC_50_ values with different letters indicate significant differences based on the relative median potency analysis of EOs against each larvae type independently.

### 3.7 Oviposition deterrence

**Figure 5:**
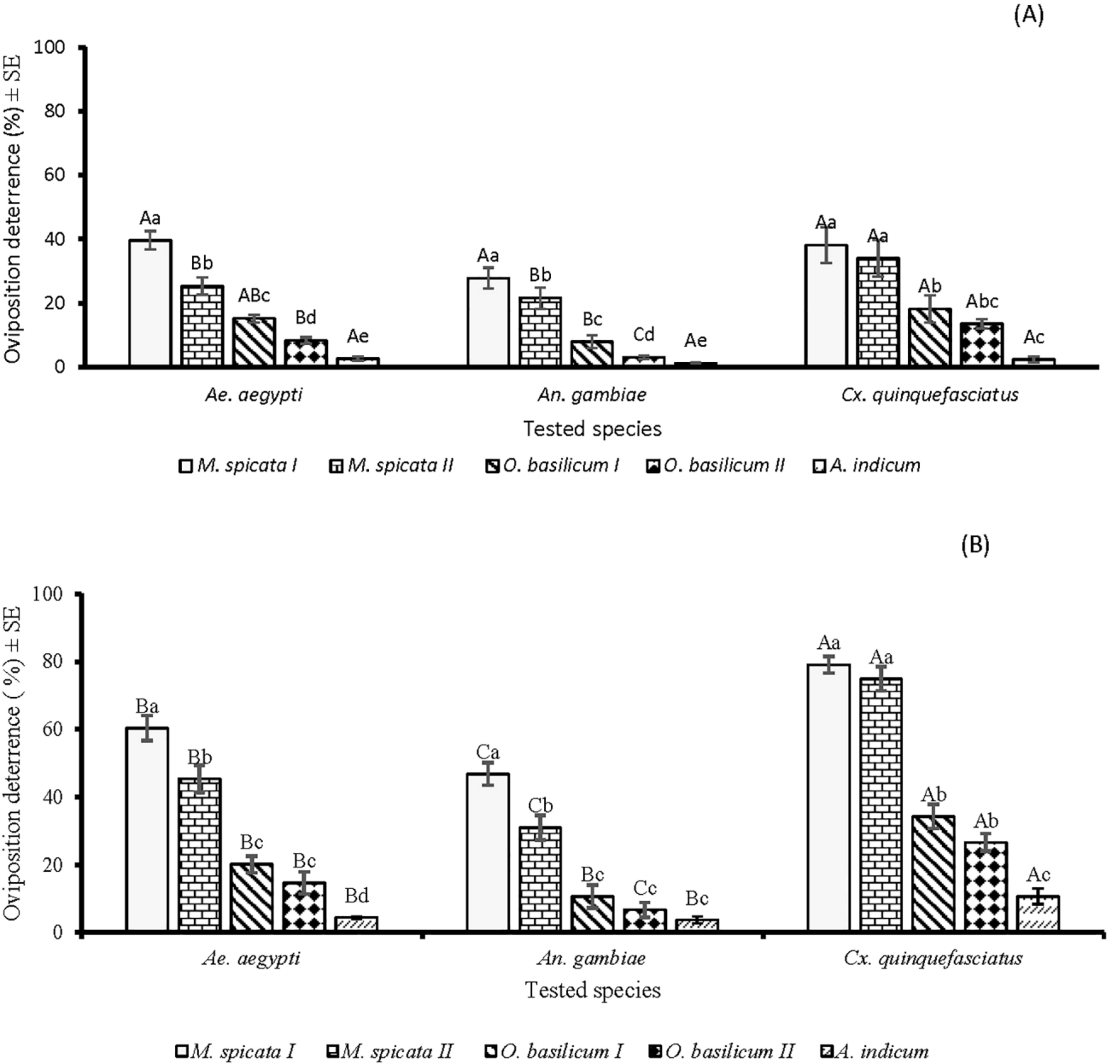
Oviposition behaviour modifying effectof five EOs at the tested doses of 60 µg/cm^2^ **(A)** and 600 µg/cm^2^ **(B)** against *Aedes aegypti*, *Anopheles gambiae* s.l., and *Culex quinquefasciatus* mosquitos. Small letters above the columns present the significant difference (*p* < 0.05) among tested EOs against mosquitoes, while capital letters present the significant difference (*p* < 0.05) among mosquitoes towards particular EO, according to ANOVA *post-hoc* Tukey test for each mosquito species and each EO separately. Error bars represent the standard error (n =5).

In the oviposition deterrence bioassay, a significant difference was observed between EOs against *Ae. aegypti* (*df* = 4, *F* = 69.5, *p* < 0.001), *An. gambiae* (*df* = 4, *F* = 20.9, *p* < 0.001), and *Cx. quinquefasciatus* (*df* = 4, *F* = 44.6, *p* < 0.001) at lower tested concentration of 60µg/cm^2^. At this concentration, higher oviposition deterrence (p < 0.05) was observed in the case of *M.* sp*icata I* against all tested species of mosquitoes compared to other tested EOs. *M.* sp*icata I* and *A. indicum* showed similar patterns of deterrence against *Ae. aegypti*, *An. gambiae*, and *Cx. quinquefasciatus* (**Figure 5A**). There was a significant difference in the oviposition deterrent effects of the tested EOs against *Ae. aegypti* (*df* = 4, *F* = 96.7, *p* < 0.001), *An. gambiae* (*df* = 4, *F* =69, *p* < 0.001), and *Cx. quinquefasciatus* (*df* = 4, *F* = 176, *p* < 0.001) at a tested concentration of 600 µg/cm^2^. EOs of *M.* sp*icata I* and *M.* sp*icata II* showed higher oviposition deterrence toward *Ae. aegypti*, *An. gambiae*, and *Cx. quinquefasciatus* as compared to the oviposition deterrent potential of *O. basilicum I*, *O. basilicum II*, and *A. indicum* EOs. Overall, greater oviposition deterrence of tested EOs was observed against *Cx. quinquefasciatus* as compared to *Ae. aegypti* and *An. gambiae* (**Figure 5B**).

### 3.8 Chemical profile of EOs

The most abundant compounds in *M.* sp*icata I* EO were piperitenone oxide (38.8%), piperitone oxide (25.6%), β-caryophyllene (6.3%), and limonene (4.1%). *M.* sp*icata II* EO contained 35.4% piperitone oxide, 22.6% piperitenone oxide, and 7.6% limonene, accounting for approximately 64.92% of its composition (**Table 4**). Estragole (55.3%), eucalyptol (10.3%), linalool (10.2), and *trans*-α-bergamotene (9%), were the most abundant compounds in *O. basilicum I*, while linalool (43.8%), estragole (14.60%), and *trans*-α-bergamotene (9%) were the major compounds in *O. basilicum II* (**Table 4**).

**Table 4:** Chemical profile of the EOs.

RI	Compounds	*M.* sp*icata I*	*M.* sp*icata II*	*O. basilicum I*	*O. basilicum II*
921	α-Thujene	0.1	0.1	tr	tr
927	α-Pinene	2.1	2.2	0.6	0.3
941	Camphene	1.3	1.2	0.1	0.1
967	Sabinene	0.9	0.9	0.5	0.3
970	β-Pinene	1.6	1.7	1.0	0.7
988	β-Myrcene	3.6	3.9	0.7	1.0
1013	α-Terpinene	0.1	0.1	tr	0.1
1020	*p*-Cymene	0.1	0.1		
1026	Limonene	4.1	7.6	0.6	0.4
1027	Eucalyptol			10.3	6.4
1036	*cis*-β-Ocimene	0.2	0.5	0.1	0.2
1046	*trans*-β-Ocimene			0.7	0.5
1056	γ-Terpinene	0.1	0.1	0.1	0.1
1063	*cis*-Sabinene hydrate	0.4	0.3	0.1	0.1
1084	Fenchone			0.3	0.2
1086	Terpinolene	0.1			0.2
1099	Linalool		0.3	10.2	43.8
1140	Camphor			0.7	0.6
1164	Borneol	3.2	2.6	0.1	0.1
1175	4-Terpineol	0.1	0.1	tr	tr
1183	*p*-Cymen-8-ol	tr	tr		
1188	α-Terpineol	tr	tr	0.5	0.6
1200	Estragole	0.3	0.2	55.3	14.8
1218	Fenchyl acetate			0.4	0.1
1241	Carvone	0.1	0.1		
1255	Piperitone oxide	25.6	35.4		
1260	*cis*-Carvone oxide	0.3			
1284	Anethole				1.6
1284	Bornyl acetate	0.6	0.8	0.3	
1285	Mintlactone	1.7	1.7		
1294	Thymol	0.4	0.9		
1298	2‐Hydroxypiperitone		0.0		
1324	Myrtenyl acetate			0.1	0.2
1336	δ-Elemene				0.1
1338	Piperitenone	0.1	0.1		
1368	Piperitenone oxide	38.8	22.6		
1384	β-Bourbonene	0.1	0.1	0.1	
1391	β-Elemene	0.2	0.3	0.8	2.3
1395	β-Cubebene			Tr	0.1
1397	*cis*-Jasmone	0.1	0.2		
1409	α-Gurjunene	1.5	2.6		
1418	β-Caryophyllene	6.3	6.2	0.5	2.7
1428	β-Gurjunene	tr	0.1		
1436	*trans*-α-Bergamotene		0.1	9.0	9.0
1446	*cis*-Muurola-3,5-diene	tr	tr	0.2	0.3
1452	α-Humulene	0.9	0.8	0.2	0.4
1458	β-Farnesene	0.3	0.2	0.2	0.2
1462	γ-Muurolene	0.6	0.6	0.3	0.7
1480	Germacrene D	2.7	3.3	0.4	2.1
1495	Elixene		0.1	0.2	1.0
1505	Eremophilene			0.2	0.6
1509	β-Bisabolene			0.1	0.1
1513	γ-Cadinene	0.1	0.2	2.0	2.3
1522	Calamenene	0.4	0.4	0.1	0.1
1524	β-Cadinene	0.2	0.2	0.2	0.3
1537	α-Cadinene	0.1	0.1	0.1	0.1
1581	Caryophyllene oxide	0.3	0.2		
1615	1,10-Di-epi-Cubenol	0.1	0.1	0.4	0.6
1641	tau-Cadinol	0.1	0.2	2.3	4.3
1655	α-Cadinol	0.1	0.1	tr	0.1

Retention index (RI) is calculated based on the retention time of C_9_-C_26_ hydrocarbons using the DB-5 gas chromatographic column. tr stands for traces having relative abundance < 0.1%.

## 4 Discussion

Plant-based products like EOs are receiving more attention due to their potential in controlling blood sucking insects, mosquitoes and ticks etc. Here, we evaluated the repellent, larvicidal, and oviposition deterrent activities of five EO samples derived from two populations of *M.* sp*icata* and *O. basilicum* as well as a population of *A. indicum* against *Ae. aegypti*, *An. gambiae*, and *Cx. quinquefasciatus* mosquitoes. Repellent results of the current study revealed that the tested EOs showed higher repellence against *Cx. quinquefasciatus* as compared to *Ae. aegypti* and *An. gambiae*. The difference in the behavioural response of different mosquito species towards the same test substance could be explained based on the presence of mismatched types of chemoreceptors in mosquito species (42). Our results are aligned with the previous studies where different levels of repellence of EOs were observed against other species of mosquitoes. For example, in a comparative study, *Cx. quinquefasciatus* was significantly more repelled compared to *Ae. aegypti* when exposed to the same EOs (42). In another study, menthol propylene glycol carbonate and DEET were significantly more repellent against *Cx. quinquefasciatus* as compared to *An. gambiae* (43). This could be due to differences in the sensitivity of olfactory receptors of different species to the chemical constituents present in the tested EOs (44).

Furthermore, the repellent activity of tested EOs against *Ae. aegypti*, *An. gambiae*, and *Cx. quinquefasciatus* was observed in a dose-dependent manner. At the higher tested dose 333 μg/cm^2^, all tested EOs showed greater and long-lasting repellence against *Ae. aegypti*, *An. gambiae*, and *Cx. quinquefasciatus* compared to the lower tested dose 33.3 μg/cm^2^. Our study results are aligned with the previous studies where the repellence of EOs has been documented as dose/concentration-dependent (35, 38, 45–47).

*M.* sp*icata I* and *M.* sp*icata II* EOs exhibited higher repellent effects against *Ae. aegypti*, *Cx. quinquefasciatus*, and *An. gambiae* as compared to the EOs of *O. basilicum I*, *O. basilicum II*, and *A. indicum*. However, in time-based bioassays, different repellent longevity of *M.* sp*icata I* and *M.* sp*icata II* was observed against *Ae. aegypti*, *An. gambiae*, and *Cx. quinquefasciatus*. The difference in repellence longevity of *M.* sp*icata I* and *M.* sp*icata II* might be due to their chemical compounds, particularly different proportions of piperitenone oxide and piperitone oxide. Previously, piperitenone oxide had displayed repellent efficacy against *An. stephensi* and *Ae. albopictus* (28, 30). In the study of Giatropoulos et al. (28) piperitenone oxide exhibited 95% repellency against *Ae. albopictus* at a tested dose of 40 μg/cm^2^. When the dose was increased to at 80 μg/cm^2^ and 200 μg/cm^2^ the repellency was 100%. Tripathi et al. demonstrated 100% repellency of piperitenone oxide against *An. stephensi* at the tested dose of 10.0 mg/mL (30). In our previous study, 45.5% piperitone oxide and 30% piperitenone oxide were the major components of the *M. longifolia* EO that showed 100% repellence against *Ae. aegypti* as compared to other tested EOs. Our current study and the previous studies showed that EOs containing a higher proportion of piperitenone oxide along with other compounds like piperitone oxide exhibited higher repellency compared to one where the relative proportion of piperitenone oxide was lower than piperitone oxide. Moreover, pure piperitenone oxide also exhibited lower repellency compared to the combined effect (28). Like in a study, though, the repellency of piperitone oxide against *Ae. albopictus* was moderate, its combined effect was significant in the case of EO, which contained 23% piperitone oxide and 41% piperitenone oxide (28). A study from India reported the presence of 32.4% piperitone oxide and 41.5% piperitenone oxide in *Plectranthus incanus* EO, which showed excellent repellency against *An. stephensi* and *Cx. fatigans* (48). Thus, the synergetic effects of different components of *M.* sp*icata I* and *M.* sp*icata II* EOs make it a potent repellent for *Ae. aegypti*, *An. gambiae*, and *Cx. quinquefasciatus.*

Previously, it has been proved that the bio-efficacy of EOs mainly depends on the type of chemical compounds present in them (49–51). In previous studies, EOs derived from *M.* sp*icata* have possessed different levels of repellency against various species of mosquitoes. For example, EO of *M.* sp*icata* possessed 73% repellence against *Ochlerotatus caspius* at a tested concentration of 20% (52). Likewise, Giatropoulos et al. from Greece reported 90% repellence of *M.* sp*icata* EO against *Ae. albopictus* at a tested dose of 40 μg/cm^2^ (28). In the current study, even at a lower tested dose of 33.3 μg/cm^2^, greater repellence of *M.* sp*icata* EO was observed against *Ae. aegypti*, *An. gambiae* and *Cx. quinquefasciatus*. The difference in the repellent effects of *M.* sp*icata* EOs evaluated in the current and previous studies might be due to differences in the chemical composition of EOs or due to the presence of different chemoreceptors in different mosquitoes. Notably, the EOs derived from *M.* sp*icata I* and *M.* sp*icata II* provided long-lasting repellency at a higher tested dose 333 μg/cm^2^ against *Cx. quinquefasciatus* even longer than the positive control, i.e., DEET. The presence of less volatile compounds like piperitenone oxide, piperitone oxide and borneol in the EO of *M.* sp*icata* might have been attributed towards the long-lasting effects of *M.* sp*icata I* and *M.* sp*icata II* EOs against the tested species of mosquito.

The EOs distilled from *O. basilicum I*, and *O. basilicum II* showed varying levels of repellency against *Ae. aegypti*, *Cx. quinquefasciatus*, and *An. gambiae*. A similar study was conducted by Baba et al., (53), where different levels of repellency of *O. basilicum* EO were observed against other species of mosquitoes (53). *O. basilicum* EO provided more repellence for a longer period (303 min) against *An. gambiae* as compared to *Cx. quinquefasciatus* (180 min) (53). In the current study, *O. basilicum I* EO showed up to 60 min repellence against *An. gambiae* while 105 min against *Cx. quinquefasciatus* at a tested dose of 333 μg/cm^2^. The difference in the repellency time of EO tested in the current study and Baba et al., (53) could be due to the different doses used, here we used 10% EO solution (333 μg/cm^2^), whereas Baba et al., (53) used pure EO having ten times higher concentration (53). Data showed the applied concentration/dose poses a significant effect on bioactivity. In the current study, the most abundant compounds of *O. basilicum* EO were estragole, eucalyptol, and linalool. These compounds, along with other compounds present in the EOs of *O. basilicum I* and *O. basilicum II*, might have contributed to the repellent activity of *O. basilicum* EOs against *Ae. aegypti*, *An. gambiae*, and *Cx. quinquefasciatus*.

In larvicidal bioassays, EOs showed varying levels of toxicity toward 2^nd^ instar larvae of *Ae. aegypti*, *Cx. quinquefasciatus*, and *An. gambiae*. *M.* sp*icata I* exhibited the highest larvicidal activity compared to all other tested EOs. The variation in the larvicidal effects of *M.* sp*icata I* and *M.* sp*icata II* might be due to the difference in the proportion of the same chemical compounds as piperitenone oxide and piperitone oxide. Previously, piperitenone oxide, which was an abundant compound (35.7%) in *M.* sp*icata* EO showed prominent larvicidal effects against larvae of *Cx. pipiens* (LC_50_ 9.95 ppm) (54). In another study, piperitenone oxide showed toxic effects against larvae of *Ae. albopictus* with an LC_50_ value of 162.2 ppm (28). In our previous study, EO having piperitone oxide (45.5%), piperitenone oxide (30.1%), and limonene (4.6%) as the most abundant compounds provided toxic effects against *Ae. aegypti* larvae (2^nd^ instar) with an LC_50_ of 39.3 ppm (35). Limonene also has been shown toxic effects against *An. stephensi*, *Ae. aegypti*, and *Cx. quinquefasciatus* with the LC_50_ values of 8.83, 12.01, and 14.07 ppm, respectively (29). So, we can say that the toxic effects of *M.* sp*icata I* and *M.* sp*icata II* against tested species of mosquitoes might be due to the presence of these compounds like piperitenone oxide, piperitone oxide, and limonene, however, the contribution of other minor compounds can also be involved in the toxic effects of *M.* sp*icata I* and *M.* sp*icata II*.

*O. basilicum I* and *O. basilicum II* showed strong toxic effects against 2^nd^ instar larvae of *Ae. aegypti*, *An. gambiae*, and *Cx. quinquefasciatus*. Our results are aligned with the previous studies where the EO of *O. basilicum* had been proven as a toxicant against various disease-carrying mosquitoes like *Ae. aegypti* (55), *Cx. quinquefasciatus* (56, 57), *Cx. tritaeniorhynchus*, *An. subpictus*, *Ae. albopictus* (37), *An. stephensi* (58), *An. culicifacies* (59), and *An. stephensi* (58). However, the toxic effect of *O. basilicum* EOs varied within the study as well as with previous studies. For example, in the current study, *O. basilicum I* and *O. basilicum II* showed different toxicity levels against *Cx. quinquefasciatus* with the LC_50_ value of 21 ppm and 61 ppm, respectively. Likewise in a previous study, EO of *O. basilicum* showed toxicity to *Cx. quinquefasciatus* larvae having an LC_50_ of 92 ppm (60). In another study, the EO of *O. basilicum* showed larvicidal activity against *Cx. quinquefasciatus* with the LC_50_ value of 68 ppm (61). The difference in larvicidal activity of *O. basilicum* EOs within the study and previous studies might be due to changes in the proportions of the same chemicals or different chemical compositions of *O. basilicum* EOs used in the current and previous studies. However, it is difficult to assess or compare results with other published data because of differences in plant sources, variation in chemical composition, percentage of secondary metabolites, extraction methods, collection times in different seasons and species of different mosquito genera (62–64). Larvicidal activity of *O. basilicum* EOs against *Ae. aegypti*, *An. gambiae*, and *Cx. quinquefasciatus* might be due to the presence of linalool and eucalyptol, which were present abundantly in the *O. basilicum* EO. Previously, both compounds have been proven to have toxic effects on the larvae of mosquitoes. For example, eucalyptol possessed 100% mortality in larvae of *Ae. aegypti* at a tested dose of 100 ppm (65). In another study, eucalyptol had toxic effects with an LC_50_ of > 200 ppm (28). Likewise, linalool displayed larvicidal activity against *Ae. aegypti* with an LC_50_ of 50 ppm (66). So, here we can conclude that chemical compounds present in EOs significantly effects the bioactivity of EO.

In current study, 2^nd^ instar larvae were used to evaluate the larvicidal activity of the tested EOs over a 48 h exposure period. This approach was chosen to avoid pupation, which could occur with 3^rd^ instar larvae during prolonged exposure. It is well-documented that younger larvae (2^nd^ instar) are more susceptible to insecticides compared to older larvae (3^rd^ or 4^th^ instar) due to their thinner cuticle, smaller size, and underdeveloped detoxification mechanisms (67–69). For instance, Rajkumar et al. (67) demonstrated that 2^nd^ instar larvae of *Ae. aegypti* exhibited significantly lower LC_50_ values when exposed to plant-derived compounds compared to 3^rd^ and 4^th^ instar larvae (67). Similarly, Chellappandian et al. (69) demonstrated that 2^nd^ instar larvae of *Ae. aegypti* exhibited significantly lower LC_50_ values when exposed to chlorpyrifos, a standard insecticide, compared to 3^rd^ and 4^th^ instar larvae (69). While this higher susceptibility and the extended exposure period may limit direct comparability with studies using 3^rd^ or 4^th^ instar larvae and a 24-hour exposure (as per WHO protocols), our findings provide valuable insights into the efficacy of the tested oils against early larval stages, which are critical targets for mosquito control programs. Future studies could include parallel experiments with 3^rd^ instar larvae and standardized exposure times to facilitate broader comparisons and further validate these results.

In oviposition deterrence bioassays, *M.* sp*icata I* and *M.* sp*icata II* EOs provided high oviposition deterrent activity against *Ae. aegypti*, *An. gambiae*, and *Cx. quinquefasciatus* as compared to the EOs of *O. basilicum I*, *O. basilicum II*, and *A. indicum*. Oviposition deterrent activity of *M.* sp*icata* EO has been documented in previous studies against mosquitoes (30). Previously, *M.* sp*icata* EO showed 47%, and 97% oviposition deterrence against *An. stephensi* at a tested dose of 30 µg/mL and 60 µg/mL, respectively (30). Chemical compounds of the *M.* sp*icata* EO might be responsible for the oviposition deterrent activity against mosquitoes. Previously, piperitenone oxide, the most abundant compound of *M.* sp*icata* EO, had displayed 77% and 100% oviposition deterrence against *An. stephensi* at 30 µg/mL and 60 µg/mL, respectively (30). Thus, the oviposition deterrent activity of the *M.* sp*icata* EO against tested species of mosquitoes might be due to the presence of major and minor compounds in it.

EO of *O. basilicum* showed significantly different levels of oviposition deterrent activity against *Cx. quinquefasciatus* (30%), *An. gambiae* (20%), and *Ae. aegypti* (10%). Our results are aligned with the previous studies where different levels of oviposition deterrence were observed against various mosquitoes. For example, *O. basilicum* EO showed 95% oviposition deterrent at varying doses against *An. stephensi* (146 μg/mL), *Ae. aegypti* (211 μg/mL), and *Cx. quinquefasciatus* (215 μg/mL) (41). In another study, *O. basilicum* EOs displayed 95% oviposition deterrence against *Ae. aegypti* at the tested concentration of 10% (70). The difference in the oviposition deterrent activity of *O. basilicum* EO within the study and with respect to previous studies can be due to a change in the chemical composition of EOs.

The most abundant compounds in *M.* sp*icata I* EO were piperitenone oxide (38.8%), piperitone oxide (25.6%), β-caryophyllene (6.3%), and limonene (4.1%). *M.* sp*icata II* EO contained 35.4% piperitone oxide, 22.6% piperitenone oxide, and 7.6% limonene. In our previous published data, the major compounds of *M.* sp*icata* EO were piperitenone oxide (47.0%), eucalyptol (12.0%), and borneol (9.5%) (24). A study by Koliopoulos et al. identified the major constituents in M. spicata as piperitenone oxide (35.7%) and 1,8-cineole (14.5%) (54). Several factors can influence the difference in the chemical composition of M. spicata EOs within the study and previous studies, including climate, altitude, soil type, growth conditions, agricultural methods and practices, plant part extracted, developmental stage, and harvesting time (71).

In the current study estragole (55.3%), eucalyptol (10.3%), linalool (10.2), and *trans*-α-bergamotene (9%) were the most abundant compounds in *O. basilicum I*, while linalool (43.8%), estragole (14.6%), and trans-α-bergamotene (9.0%) were the major compounds in *O. basilicum II* EO. Previously, linalool (56.7–60.6%) was the main constituent of *O. basilicum* EO, followed by *epi*-α-cadinol (8.6–11.4%), α-bergamotene (7.4–9.2%), γ-cadinene (3.3–5.4%) (72). Purkayastha et al. reported camphor, limonene and β-selinene as the major compounds of *O. basilicum* EO (73). The observed difference in the chemical composition of *O. basilicum* EOs within the current study and with respect to the previous studies might be due to the presence of different chemotypes, soil types and availability of nutrients to plants. Previously, *O. basilicum* EO exhibited a wide and varying array of chemical compounds, depending on variations in chemotypes, leaf and flower colours, aroma and origin of the plants (72, 74, 75).

The industrial application of essential oil-based formulations as mosquito repellents holds promise due to their natural origin and lower environmental impact. However, challenges such as formulation stability, volatility, and cost-effective large-scale production must be addressed. While essential oils offer a sustainable alternative to DEET, their commercial viability depends on optimizing extraction processes, enhancing longevity through encapsulation techniques, and ensuring affordability for widespread consumer adoption.

## Conclusions

The use of EOs in controlling mosquitoes is expected to reduce the cost and harmful environmental effects of synthetic mosquito control measures. *M.* sp*icata I* EO proved effective as a repellent, larvicidal, and oviposition deterrent against *Ae. aegypti*, *An. gambiae*, and *Cx. quinquefasciatus*. In the time span bioassay, *M.* sp*icata I* EO showed significantly higher repellence than the “golden repellent standard” DDET against *Ae. aegypti* and *Cx. quinquefasciatus*; therefore, this EO can be considered a potential candidate for controlling mosquitoes. The limited repellent, larvicidal, and oviposition deterrent efficacy of *A. indicum* EO reveals no potential of this formulation for mosquito management. Further research should focus on optimizing the formulation of *M.* sp*icata* I EO for field applications, including microencapsulation or emulsification techniques to enhance stability and longevity.

## Data Availability

The raw data supporting the conclusions of this article will be made available by the authors, without undue reservation.
